# Enzymatic Metabolic Switches of Astrocyte Response to Lipotoxicity as Potential Therapeutic Targets for Nervous System Diseases

**DOI:** 10.3390/ph17050648

**Published:** 2024-05-16

**Authors:** Andrea Angarita-Rodríguez, J. Manuel Matiz-González, Andrés Pinzón, Andrés Felipe Aristizabal, David Ramírez, George E. Barreto, Janneth González

**Affiliations:** 1 Departamento de Nutrición y Bioquímica, Facultad de Ciencias, Pontificia Universidad Javeriana, Bogotá 110231, Colombia; 2 Laboratorio de Bioinformática y Biología de Sistemas, Universidad Nacional de Colombia, Bogotá 111321, Colombia; 3 Molecular Genetics and Antimicrobial Resistance Unit, Universidad El Bosque, Bogotá 110121, Colombia; 4 Departamento de Farmacología, Facultad de Ciencias Biológicas, Universidad de Concepción, Concepción 4030000, Chile; 5 Department of Biological Sciences, University of Limerick, V94 T9PX Limerick, Ireland; 6 Health Research Institute, University of Limerick, V94 T9PX Limerick, Ireland

**Keywords:** astrocytes, metabolic switches, drug-protein network, druggable cavity, therapeutic targets

## Abstract

Astrocytes play a pivotal role in maintaining brain homeostasis. Recent research has highlighted the significance of palmitic acid (PA) in triggering pro-inflammatory pathways contributing to neurotoxicity. Furthermore, Genomic-scale metabolic models and control theory have revealed that metabolic switches (MSs) are metabolic pathway regulators by potentially exacerbating neurotoxicity, thereby offering promising therapeutic targets. Herein, we characterized these enzymatic MSs in silico as potential therapeutic targets, employing protein–protein and drug–protein interaction networks alongside structural characterization techniques. Our findings indicate that five MSs (P00558, P04406, Q08426, P09110, and O76062) were functionally linked to nervous system drug targets and may be indirectly regulated by specific neurological drugs, some of which exhibit polypharmacological potential (e.g., Trifluperidol, Trifluoperazine, Disulfiram, and Haloperidol). Furthermore, four MSs (P00558, P04406, Q08426, and P09110) feature ligand-binding or allosteric cavities with druggable potential. Our results advocate for a focused exploration of P00558 (phosphoglycerate kinase 1), P04406 (glyceraldehyde-3-phosphate dehydrogenase), Q08426 (peroxisomal bifunctional enzyme, enoyl-CoA hydratase, and 3-hydroxyacyl CoA dehydrogenase), P09110 (peroxisomal 3-ketoacyl-CoA thiolase), and O76062 (Delta(14)-sterol reductase) as promising targets for the development or repurposing of pharmacological compounds, which could have the potential to modulate lipotoxic-altered metabolic pathways, offering new avenues for the treatment of related human diseases such as neurological diseases.

## 1. Introduction

Astrocytes are specialized glial cells that play a crucial role in various intricate processes within the brain. They are pivotal in the maintenance of a healthy central nervous system and the management of pathological conditions. Their functions include elimination of toxic substances, synaptogenesis, neurotransmitter release, and modulation of neuroinflammation, among others [1,2,3,4,5]. Astrocytes perform various essential functions for brain homeostasis and neuronal function [6]. These cells regulate glutamate and ion homeostasis, cholesterol, and sphingolipid metabolism, and respond to environmental factors exerting neuroprotective effects maintaining general homeostasis of the nervous system [7]. Therefore, one of the crucial functions of astrocytes is their involvement in fatty acid metabolism [8]. Recent research has highlighted the significance of palmitic acid (PA), a long-chain saturated fatty acid that has been found to induce proinflammatory effects and reduce astrocyte viability, a condition known as lipotoxicity. This scenario is characterized by the accumulation of excessive lipids and the subsequent generation of lipotoxic molecules, leading to impaired astrocyte function and viability, which can contribute to neuroinflammation and neuronal damage. In this context, it has been observed that PA triggers proinflammatory signaling pathways, such as the NF-kB pathway, resulting in the upregulation of proinflammatory cytokines, such as TNF, IL-1, and IL-6 [9,10,11]. These cytokines disrupt cellular metabolism and elicit inflammatory responses, ceramide formation, and oxidative stress [11,12,13].

The intricate interplay between lipotoxicity and cellular dysfunction has garnered significant attention, particularly in various metabolic disorders, including neurodegenerative diseases [14]. Saturated fatty acids, such as palmitic acid (PA), are closely associated with the development of conditions such as dementia, amyotrophic lateral sclerosis (ALS), Alzheimer’s disease (AD), and Parkinson’s disease (PD) [15]. While recent studies on astrocyte metabolism under lipotoxicity have primarily concentrated on deciphering specific elements through experimental simulations, there has been a notable gap in comprehending the systemic mechanisms operating at various organizational levels. This lack of understanding of the metabolic relationships in pathological conditions has prompted the need for more holistic strategies. To obtain a more comprehensive perspective on the cellular functions of brain behavior, genome-wide metabolic networks (GEM) and control theory have been employed. These innovative approaches have enabled the identification of reaction groups that exert direct or indirect control over cellular metabolism, even in the absence of prior knowledge of specific cellular targets at the in silico level [16]. Consequently, the mathematical modeling of metabolic processes has facilitated the identification of crucial control sites and their contribution to the overall functionality of a given metabolic network. According to the most recent study conducted by our team [8], we identified 16 enzymatic metabolic switches in astrocytes treated with PA. Enzymatic metabolic switches (MSs) serve as critical control points, facilitating transitions between metabolic states and allowing cells to adapt to varying environmental cues and physiological demands. These switches play a fundamental role in both the regulation and deregulation of metabolic pathways in lipotoxic conditions, intensifying neurotoxicity, and potentially serving as valuable treatment targets.

Due to their relevance, metabolic switches (MSs) could be evaluated as potential targets of drugs and drug-like small molecules approved by the US Food and Drug Administration (FDA). However, it is necessary to determine the pharmacological features that clarify the potential of these enzymes to be proposed as therapeutic targets of pathological conditions associated with lipotoxicity. Structural-based computational analyses of protein-binding sites have been used to predict the pharmacological potential by transforming structural and physicochemical properties (hydrogen bonding, hydrophobicity, polarity, and amino acid composition, among others) into quantitative metrics of druggability [17]. In addition, biological network analyses, such as the protein–protein interactions network (PPI) and the drug–protein interactions network (DPI), can be useful for organizing and analyzing biological data [18,19] and promoting the analysis of biological information from experimental databases to elucidate new conclusions on new potential drug–target interactions [19,20,21] or polypharmacological activities [19,21].

This study aimed to elucidate the potential of enzymatic MSs of the human astrocyte response to lipotoxicity as therapeutic targets for related diseases, such as nervous system diseases. Using a multidisciplinary approach integrating different in silico methods (network analysis and structural bioinformatics), this research seeks to uncover novel targets within the lipotoxic cascade that could be harnessed to mitigate astrocyte-mediated neurodegenerative processes, promoting the study of innovative therapeutic molecules for human neurodegenerative disorders.

## 2. Results and Discussion

### 2.1. Enzymatic Metabolic Switches (MSs) Are Functionally Related to Metabolic Pathways That ARE altered under Neurodegenerative Conditions

**Figure 1:**
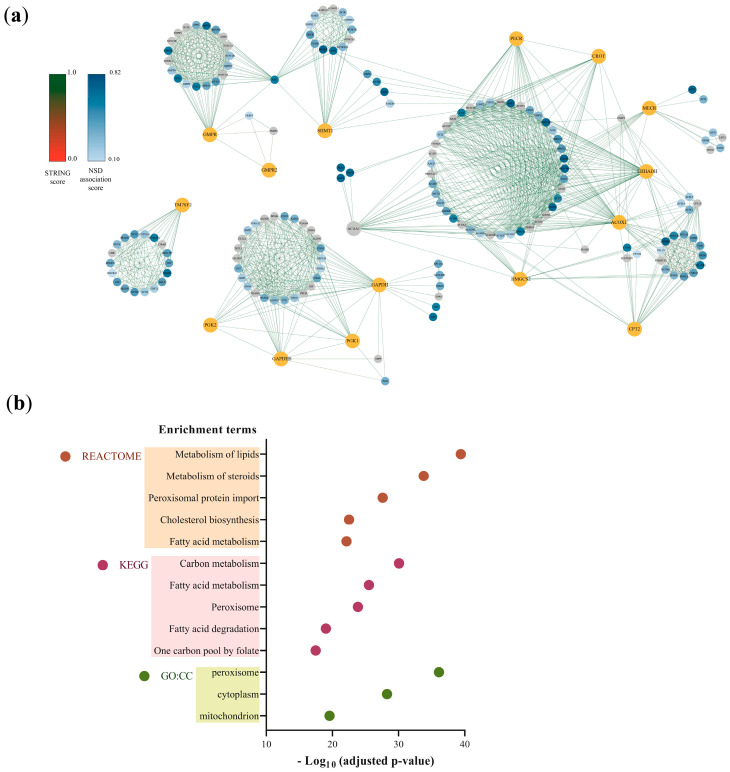
(**a**) Protein–protein interaction (PPI) network between metabolic switches and their STRING first neighbors. Metabolic switches are shown in orange, and STRING first neighbors (N1) are shown on a blue scale according to their nervous system disease (NSD) association score (score from 0.1 to 1.0) obtained from the Open Target Database. Those N1 with little or no association (<0.1) are shown in gray. (**b**) Enriched terms in proteins functionally related to metabolic switches, obtained from Gene Ontology Cellular Component (GO:CC), KEGG, and REACTOME databases. The adjusted *p*-value reflects the statistical significance of each term’s enrichment within the set of proteins associated with MSs.

To identify whether any of the metabolic switches have the potential to be targets in neurological diseases, we established a functional relationship between these MSs and drug targets associated with neurological diseases. Initially, our analysis revealed that the 16 MSs are connected to 175 proteins (Figure 1a), most of these being reported as nervous system disease targets by the Open Target database, and these functional interactions were well supported according to the different interaction sources employed by STRING (text mining, experiments, databases, co-expression, neighborhood, gene fusion, and co-occurrence). These proteins participate in enriched metabolic pathways, such as fatty acid catabolism and the one-carbon folate cycle, and are present in cellular components, such as mitochondria and peroxisomes (Figure 1b) (Table S1 in the Supplementary Materials). Notably, these metabolic pathways are also known to be (de)regulated in human astrocytes under lipotoxic conditions because of the control exerted by these MSs [8]. Furthermore, previous studies have linked alterations in mitochondrial and peroxisomal fatty acid oxidation, along with other functions in these organelles, to the development of neuroinflammation and neurodegeneration [22,23,24,25].

### 2.2. Trifluperidol, Trifluoperazine, Disulfiram, and Haloperidol Would Significantly Affect MSs

**Figure 2:**
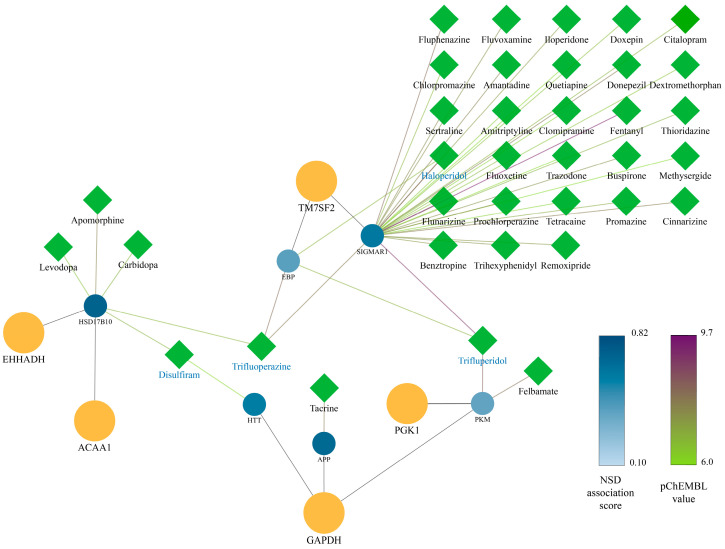
DPI network. Metabolic switches are shown as orange circles and their first neighbors as circles on a blue scale according to their association score for nervous system diseases (NSD) obtained from the Open Target Database. Edges between nervous system phase 4 drugs (green diamonds) with first neighbors are colored on a green–violet scale according to their pChEMBL interaction score. Drug names colored in blue correspond to molecules with more than one target in the DPI network.

After establishing the proteins functionally associated with MSs, we generated a drug–target interaction network (DPI), integrating the approved neurological drugs previously reported for proteins in the PPI network. Interestingly, none of the drugs were found to directly target a metabolic switch, which supports the novelty of MSs as therapeutic targets of nervous system diseases. In the DPI, we identified 37 drugs that interact with proteins functionally related to five metabolic switches (Figure 2), which may be modulating these MSs indirectly during their action. A search in OpenTargetsDB showed that two of these five proteins (PGK1: P00558, and GAPDH: P04406) are highly associated with neurodegenerative diseases according to the association score for this neurological condition, supported primarily by Pathways System Biology data (Table S2). By contrast, EHHADH (Q08426), ACAA1 (P09110), and TM7SF2 (O76062) presented little or no association with these diseases (Table S2), which argues for their novelty as proteins potentially related to neurodegenerative diseases.

Finally, to identify the drugs with the greatest impact on the MSs, we determine the drugs with multiple PPI targets, finding that four of these drugs (CHEMBL15023: Trifluperidol, CHEMBL422: Trifluoperazine, CHEMBL964: Disulfiram, CHEMBL54: Haloperidol) have polypharmacological potential over MS-related proteins (Table 1). The 33 drugs with a single target in the DPI network are described in Table S3 in the Supplementary Materials.

Trifluperidol (CHEMBL15023) has had three targets (PKM, EBP, and SIGMAR1) that are functionally related to the three MSs (PGK1, GAPDH, and TM7SF2) (Table 1). Thus, the pharmacological activity of trifluperidol may be associated with the modulation of glycolysis and cholesterol biosynthesis [26,27]. This drug has been widely used for NR1a/2B receptors and is indicated for treating schizophrenia, behavioral disorders, motor disorders, and edema in psychotic illnesses [28]. Trifluperidol has preclinical evidence of neuroprotective properties by promoting the reduction of tumor necrosis factor-alpha (TNF-α) and nitric oxide secretion in microglial cells [29] and modulating proinflammatory cytokines (IL- 1-beta and IL-2) in glial cells [30]. Furthermore, the modulatory nature of trifluperidol contains inhibitory characteristics. For example, the modulation of TNF-α can have an impact on neurodegenerative processes associated with demyelination and degeneration, as it induces the production of growth-promoting cytokines and neurotrophins, as well as nitric oxide and free radicals, among others that favor apoptotic processes [31,32,33]. Although most studies have described that the energy demand in patients with processes involving the CNS increases, there is a lack of studies that describe the metabolic effect of drugs such as trifluperidol. However, it has been described that antipsychotic drugs managed to improve the enzymatic activity associated with the glycolysis pathway [34].

Trifluoperazine (CHEMBL422) has three targets (EBP, HSD17B10, and SIGMAR1) that are functionally related to the three MSs (TM7SF2, ACAA1, and EHHADH) (Table 1). Thus, the pharmacological activity of trifluoperazine may be associated with the modulation of fatty acid oxidation, cholesterol biosynthesis, and amino acid catabolism [26,35,36]. It is an antipsychotic drug that inhibits dopamine receptor type 2 (DR2) [37]. Trifluoperazine suppresses the release of cytokines and exerts anticancer effects [38]. Recently, trifluoperazine has been observed to reduce the levels of interleukin-1-beta and TNF-alpha, attenuating the production of proinflammatory mediators [39,40]. Huang et al., (2021) have described that trifluoperazine reduces hypothalamic inflammation in the acute stage by suppressing gliosis induced by saturated fatty acids and inhibiting the protein calmodulin, which binds to Ca+2 in edema brain processes [39,41]. Therefore, this drug modulates metabolic dysfunction, where it has been described that its administration has a positive effect on the homeostasis of the glucose pathway [39,42,43]. Likewise, trifluoperazine has been shown to rescue dopaminergic cells and reduce a particular species of alpha-synuclein, a protein involved in PD [37]. In multiple sclerosis, the administration of trifluoperazine improves deficiencies in motor coordination and demyelination processes [44].

Disulfiram (CHEMBL964) has two targets (HSD17B10 and HTT) functionally related to three MSs (ACAA1, EHHADH, and GAPDH) (Table 1). Thus, the pharmacological activity of disulfiram could be associated with glycolysis and fatty acid oxidation modulation [35,45]. It has been widely used as an anti-alcoholic drug for relapse prevention [46]. Disulfiram has shown anti-inflammatory and neuroprotective effects [47]. For example, it decreases the activity of the BACE-1 promoter and increases the activity of ADAM10 [47,48,49]. Therefore, Disulfiram could be a therapeutic strategy by increasing the amount of the sAAP-alpha protein, which exerts neurotrophic and neuroprotective properties, since authors such as Reinhardt et al., (2018) have shown that the sAAP-alpha protein protects primary neurons in the hippocampus to apoptosis via the AKT survival pathway. In addition, studies carried out in mice have shown that disulfiram increases functional behavior, influencing AD pathology [47,50]. However, predicting the beneficial results of disulfiram remains difficult, considering the few clinical studies.

Haloperidol (CHEMBL54) has two targets (EBP and SIGMAR1) that are functionally related to a single MS (TM7SF2) (Table 1). Thus, the pharmacological activity of haloperidol could be associated with the modulation of lipid metabolism [26]. This antipsychotic drug is used for the treatment of schizophrenic symptoms, such as the control of delusions and hallucinations, and behavioral symptoms associated with Alzheimer’s disease [51]. However, the use of haloperidol is limited by its association with extrapyramidal symptoms, such as increased memory loss in patients with AD and Parkinsonism due to dopamine-receptor blockade, which leads to the generation of reactive oxygen species (ROS) [52,53]. Thus, haloperidol causes deterioration in the storage of antioxidant enzymes by increasing oxidative stress and lipid peroxidation, which is a causal factor of neurodegeneration [51,53].

Herein, we demonstrated that five MSs (TM7SF2: Delta(14)-sterol reductase; PGK1: phosphoglycerate kinase 1; GAPDH: glyceraldehyde-3-phosphate dehydrogenase; ACAA1: peroxisomal 3-ketoacyl-CoA thiolase; and EHHADH: peroxisomal bifunctional enzyme) are functionally related to neuropathological drug targets, suggesting that these MSs could be indirectly modulated as part of drug action. In addition, some of these drugs act on multiple targets related to MSs, suggesting polypharmacological activity. Most neurodegenerative diseases have a multifactorial nature, which, from the perspective of drug development, has raised questions about the classic paradigm of “one drug, one target, one disease” [54,55]. However, a holistic vision has allowed us to observe the multiplicity of the underlying pathways involved in neurodegenerative processes [56,57]. Polypharmacology has received recognition because a single drug approved by the FDA can exert a therapeutic effect on multiple targets linked to different disease pathways [56,58]. However, they may have neuroprotective properties, and their long-term use carries potential risks. More research is needed to fully understand the relationship between these medications and neurodegeneration.

Although most of the medications found are related to psychiatric diseases, psychiatric and neurodegenerative diseases often share some common biological pathways, such as inflammation, oxidative stress, and dysfunctions in neuronal signaling [31]. Several studies have investigated the relationship between these two therapeutic areas and found that they share epidemiological and genetic risk factors and distinct and shared causal proteins [59]. For example, schizophrenia and Parkinson’s diseases share common biological pathways through dopaminergic and synaptic dysfunction and neuroplasticity [60]. Furthermore, excitotoxicity appears to be the final common pathway of many neuropsychiatric and neurodegenerative disorders [59]. Moreover, some studies suggest that antipsychotics may have both beneficial and harmful effects in patients with neurodegenerative disorders. For example, atypical antipsychotics, such as risperidone, have been used to treat psychiatric symptoms associated with Alzheimer’s disease. However, concerns have also been raised about possible adverse effects, such as increased risk of stroke and the decline in cognitive function in this population [61]. In addition, Trifluoperazine, one of the antipsychotic drugs characterized as potential drugs for MSs, exhibits promise in reducing demyelination in multiple sclerosis (MS) and suppressing inflammation in the brain induced by a high-fat diet, suggesting potential therapeutic benefits for neurodegenerative diseases [39]. However, it may also induce cell death in mitochondria, indicating potential cytotoxic effects. Further research is needed to understand fully its benefits and risks in treating neurodegenerative diseases and other conditions.

It has been found that medications such as disulfiram, a drug indicated for chronic alcoholism treatment, are converted to an active metabolite, diethyldithiocarbamate, which has been shown to have potential neuroprotective effects by modulating secretase activity [47]. Furthermore, disulfiram has been shown to suppress the activation of the NLRP3 inflammasome, which is involved in neuroinflammation [62]. Finally, a recent study identified disulfiram as a potent activator of DJ-1, which has been proposed as a potential therapeutic target against neurodegenerative diseases [63]. While available research suggests that disulfiram may have potential therapeutic effects on neurodegeneration, further research or specific studies on this topic may be needed to establish a comprehensive understanding of the potential impact of disulfiram on neurodegeneration.

The reported targets for Trifluperidol, Trifluoperazine, Disulfiram, and Haloperidol functionally interact with the MSs. We consider that the MSs may be indirectly modulated by the four drugs as part of their pharmacological mechanism. This premise highlights these interactions between drug targets and the MSs, as these events are potentially regulated through drugs for nervous system diseases. Therefore, there are metabolic pathways to explore given their possible implication in the development of nervous system diseases, including diseases treated by these drugs (psychiatric diseases) or related to MSs (neurodegenerative diseases).

### 2.3. Enzymatic Metabolic Switches Related to Nervous System Drugs Have Interesting Structural Characteristics with Druggable Potential

In recent decades, methods based on structural geometry, analysis of the surface and volume of protein cavities, surface area, and the ability to form hydrogen bonds [64,65,66] have enabled the identification of the structural characteristics of proteins that promote ligand recognition. Therefore, considering the previous results, we carried out an in silico characterization of the druggable cavities of the five MSs associated with neurological drugs through ligandability and druggability scores for each enzyme and its respective natural ligand (Table S4 in the Supplementary Materials).

To perform druggable cavity analysis, ligand and orthosteric binding sites were obtained for the five switches of interest (Table 2). Four of the MSs analyzed had druggable cavities in their active sites, with acceptable ligandability (Pred. Max pKd from 9.48 to 11.39) and druggability scores (Drug score from 656 to 3753) (Table 2). Thus, they were considered to have pharmacological potential. Only one of the MSs analyzed (Delta(14)-sterol reductase TM7SF2) does not have druggable cavities, so it was ruled out as a possible directed target. The detection, characterization, and analysis of cavities allowed us to predict potential binding sites of covalent ligands (drugs), promoting the study and design of drugs with the potential to interact with metabolic switches [67,68].

**Figure 3:**
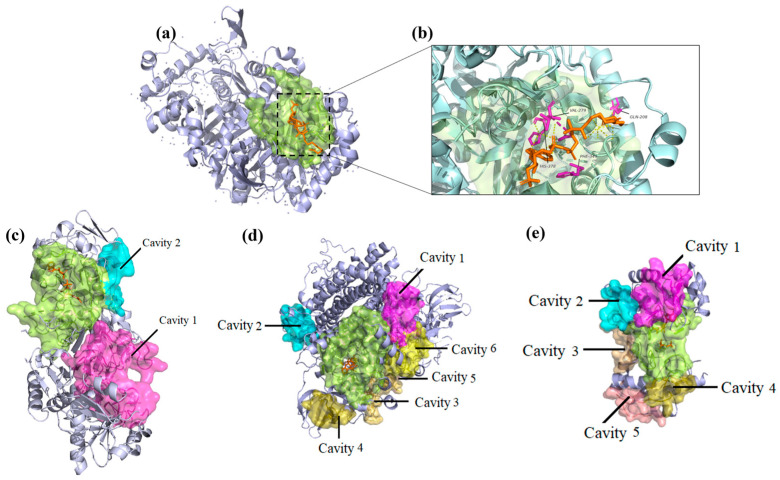
Tertiary structure and druggable cavities predicted in four of the five MSs related to nervous system drug targets. (**a**) The druggable cavity identified in the ligand-binding site of 3-ketoacyl-CoA thiolase (green) that catalyzes the condensation reaction of acyl-CoA or acyl-acyl ACP with malonyl-CoA to form 3-ketoacyl-CoA in the presence of the ligand Acetyl coenzyme A (orange). The surface represents the monomeric structure of the enzyme 3-ketoacyl-CoA thiolase (Uniprot ID: P09110). (**b**) Magnified view of the interactions between the ligand and binding amino acids (Gln208, Ser277, Val279, Phe349, and His378). (**c**–**e**) Druggable cavities predicted in glyceraldehyde-3-phosphate dehydrogenase (Uniprot ID: O14556), peroxisomal bifunctional enzyme (Uniprot ID: Q08426), and phosphoglycerate kinase 1 (Uniprot ID: O14556), respectively. Predicted druggable cavities in the ligand-binding site are colored green. Non-green cavities are allosteric cavities with druggable potential predicted for the ligand-binding site.

It is important to emphasize the role of MSs in processes such as glycolysis/gluconeogenesis, folate metabolism, fatty acid oxidation, purine catabolism, peroxisomal transport, and extracellular transport (Table S5 in the Supplementary Materials). The capacity to inhibit or activate enzymes associated with fatty acid oxidation has been studied in neurodegenerative contexts. The enzyme 3-ketoacyl-CoA thiolase (ACAA1, EC:2.3. 1.9; Uniprot ID: P09110) has been extensively studied for treatment with trimetazidine to improve insulin resistance in obese mice experimentally [69,70]. In addition, recent studies have shown the positive effect of Trimetazidine and Progesterone on brain injury [71]. Furthermore, multiple interactions (alkyl, pi-sigma, and pi-cation) have been described between Trimetazidine/Progesterone and the amino acids Ser, Val, and Phe that are present in the active site of the molecular targets 3-ketoacyl-CoA thiolase (UniProt ID: P09110) and enoyl reductase (UniProt ID: Q9BV79) [69]. Interestingly, ligand-binding pockets predicted in 3-ketoacyl-CoA thiolase (Figure 3a,b) showed similar interactions with the predicted ligand. The interaction of this enzyme with these compounds is an example of the ligand-binding ability of the druggable cavities identified in 3-ketoacyl-CoA thiolase, confirming its potential as a drug receptor.

Regarding the enzyme glyceraldehyde 3-phosphate dehydrogenase (GAPDH, EC 0.1.2.1.12; UniProt ID P04406), we find a cavity with a strongly druggable potential that also could be potentially regulated by two identified allosteric druggable cavities (Table 2, Figure 3c,d). Previous studies determined that the interaction of this enzyme with alpha-synuclein in Lewy bodies and the GAPDH active site, specifically with cysteine residues, modulates alpha-synuclein aggregation in PD [72,73]. However, studies describe the allosteric regulation of the channeling of substrates in the active site of this enzyme through the assembly of oligomers and the NAD binding site [72,73]. In addition, previous studies identified that Ser 50 and Tyr 41 play an important role in enzyme stabilization and, thus, in the regulation of metabolism by interacting the substrate with GAPDH through allosteric regulation by differences in enzyme–substrate deactivation rates between different substrates or isoenzymes [72].

According to the multi-omic computational model developed by [8], in the presence of PA, reactions such as the catalysis of the reversible transfer of a phosphate group from 1,3-bisphosphoglycerate to ADP-producing 3-phosphoglycerate and ATP (PGK, EC. 2.7.2.3; UniProt ID: P00558) by phosphoglycerate kinase are partially inhibited [8]. At the level of the active site, this enzyme presents a cavity with strong druggable potential, thus being a potential therapeutic target. In this regard, recent studies have determined that the yeast cell cycle regulatory kinase inhibitor NG52, when coupled to the active site of PGK, decreases the phosphorylation of proteins such as pyruvate dehydrogenase kinase 1, potentially inhibiting glioma proliferation through the pharmacological regulation of PGK [74,75]. As far as we know, peroxisomal bifunctional enzymes (EHHADH, EC. 1.3.3.6; Uniprot ID: Q08426) have not been previously suggested as pharmacological targets in any neuropathological context. Then, it could be a very novel target with therapeutic potential for pathologies promoted by lipotoxicity conditions. Ligand-binding and allosteric druggable cavities identified for PGK and EHHADH are modeled in Figure 3.

Although in silico studies are valuable for exploring variables, designing drugs, and reducing reliance on animal experimentation, validation with experimental data is crucial to ensure accuracy and biological relevance, enhancing their utility in scientific research. For instance, recent research in ACAA1, one of the MSs proposed as potential targets for nervous system diseases, has focused on its interactions with the β-oxidation pathway and their potential role in neurodegeneration. In the central nervous system, ACAA1 is vital for fatty acid metabolism. Deficiencies can lead to neurodegenerative disorders [76]. Experimental evidence [76] highlights ACAA1’s role in regulating intracellular Ca2+ levels in neurodegenerative diseases, interacting with IP3R and SERCA, potentially impacting conditions like Alzheimer’s and Parkinson’s disease.

This study explores potential therapeutic targets for neurological diseases associated with astrocyte-mediated neurotoxicity, focusing on pro-inflammatory pathways triggered by palmitic acid. Despite these exciting findings, the research is mainly based on in silico characterizations of MSs associated with the human astrocyte lipotoxicity response; therefore, this study does not characterize a single type of disease. Thus, we propose experimental validation in the future to determine the applicability and address challenges associated with translating computational predictions into effective therapeutic interventions for nervous system disorders.

## 3. Materials and Methods

### 3.1. Metabolic Switches Analyzed in This Study

The enzymatic metabolic switches analyzed in this study were identified in a previous study [8]. A description of all previously reported MSs can be found in Table S5 of the Supplementary Materials.

### 3.2. Protein–Protein Interactions Network (PPI)

In this study, we constructed a comprehensive map of MS-associated protein interactions, using the Knime analytics platform v4.3.0 [77] to obtain human PPI for each enzymatic metabolic switch (MS) from the STRING database [78]. Only the first shell (N1) with a medium confidence interaction score (>0.400) according to all STRING interaction sources, was used to construct an MS-PPI network using Cytoscape software v3.8.2 [79]. Then, N1 proteins were subjected to an enrichment analysis of the KEGG and REACTOME pathways, as well as gene ontology terms for cellular components (GO:CC), using the g:Profile server [80]. MS–MS interactions were also extracted from the STRING database. N1 proteins were searched in the Open Targets database [81] to retrieve the highest mean association score for any nervous system disease.

### 3.3. Drug–Protein Interactions Network (DPI)

We developed a pipeline utilizing Knime to identify compounds with activity against protein–protein interactions (PPIs), applying a specified pChEMBL value threshold [82]. This information was sourced from the ChEMBL database (release: ChEMBL31, last accessed on 21 November 2022) [83]. Our selection of drugs adhered to certain criteria, specifically those categorized as “small molecules” with information on “max phase” (0, 1, 2, 3, and 4). In cases where a drug had multiple pChEMBL values against a biological target, we considered the highest reported value. Subsequently, we filtered the data to focus on approved neurological drugs (in Phase = 4) that interacted with the identified targets. Finally, we harnessed the refined dataset to construct a drug–protein interaction (DPI) network using Cytoscape. The DPI-network MSs were searched in the Open Targets database [81] to retrieve all associated nervous system diseases.

### 3.4. Targets 3D Structures and Associated Ligands

The 3D structures of the MSs functionally related to neurological drug targets were retrieved from the PDB (Protein Data Bank) database [84]. Any experimental structure that met the following criteria was considered: (1) 80% coverage, (2) high resolution (<2.6 Å), and (3) no fragmentation. Proteins without a PDB structure were obtained from the AlphaFold database [85].

The natural ligands of each protein were identified by reviewing the enzymatic reaction of each protein in the UniProt and Mechanism and Catalytic Site Atlas databases [86]. The natural ligand is any molecule modified by the activity of the protein; if there is no natural ligand, a cofactor is selected. The natural ligands and their binding sites were obtained from the crystallographic complexes reported in the PDB. Proteins without co-crystallized ligands were analyzed using the COACH-D server [87]. In the COACH-D server, potential ligands and their binding sites were predicted using crystallographic complexes of related proteins, and the coupling between the ligand and the predicted binding site was refined using AutoDock Vina. [88].

The protein and ligand data employed in this analysis are summarized in Table S4 in the Supplementary Material.

### 3.5. Characterization of Cavities with Druggable Potential

To characterize cavities associated with the ligand-binding sites of MSs, each MS and its natural ligand were characterized in the CavityPlus server using the “With Ligand” option [67]. In addition, we predicted potential allosteric binding sites using the “No Ligand” option of the Cavity tool and the CorrSite2.0 tool, both available from the same server [67]. In this context, an allosterically regulated pocket was defined as any pocket with a CorrSite value exceeding 0.5, indicating a correlation with the ligand-binding site pocket. Then, we gauged the druggable potential of the identified cavities by assessing their ligandability (Pred. Max pKd) and druggability (DrugScore) through the CavPharmer tool from the CavityPlus server. We considered any cavity with a Pred. Max pKd exceeding 6.0 and possessing a medium or strong DrugScore as a cavity with druggable potential. Finally, the druggable cavities were visualized using the PyMOL molecular graphics system, version 2.5 (Schrödinger, 2015).

## 4. Conclusions

Neurodegenerative diseases are complex, with multiple cellular and molecular mechanisms at play. It is crucial to adopt a holistic approach when studying the pathological conditions that underlie these diseases, such as the impact of palmitic acid (PA) on astrocytes, which is associated with lipotoxicity. This comprehensive understanding is vital for the development of effective treatments. In our study, we concentrated on the identification and characterization of potential pharmacological targets within the enzymatic metabolic switches (MSs). These MSs had been previously linked to metabolic alterations in response to PA in astrocytes. Through this approach, we pinpointed five enzymatic MSs (P00558: phosphoglycerate kinase 1, P04406: glyceraldehyde-3-phosphate dehydrogenase, Q08426: peroxisomal bifunctional enzyme—enoyl-CoA hydratase and 3-hydroxyacyl CoA dehydrogenase, P09110: peroxisomal 3-ketoacyl-CoA thiolase, and O76062: Delta(14)-sterol reductase) that exhibited functional connections to the drug targets associated with neurological conditions. Moreover, the first four of these MSs featured potentially druggable cavities linked to their ligand-binding sites. Consequently, we propose phosphoglycerate kinase 1, glyceraldehyde-3-phosphate dehydrogenase, peroxisomal bifunctional enzyme—enoyl-CoA hydratase and 3-hydroxyacyl CoA dehydrogenase, and peroxisomal 3-ketoacyl-CoA thiolase as promising therapeutic targets for neurological conditions.

## Figures and Tables

**Table 1:** Nervous-system drugs identified in the DPI network with potential polypharmacological potential against MSs.

Drug Name (CHEMBL ID)	Drug Group	Pharmacological Activity	Number of Targets	ID UniProt Target	Targets	Metabolic Switch Related
**Trifluperidol** (CHEMBL15023)	Butyrophenone derivatives	Modulation of glycolysis and cholesterol biosynthesis	3	P14618	PKM	PGK1, GAPDH
				Q15125	EBP	TM7SF2
				Q99720	SIGMAR1	TM7SF2
**Trifluoperazine** (CHEMBL422)	Phenothiazines with piperazine structure	Modulation of fatty acid oxidation, cholesterol biosynthesis, and amino-acid catabolism	3	Q15125	EBP	TM7SF2
				Q99714	HSD17B10	ACAA1, EHHADH
				Q99720	SIGMAR1	TM7SF2
**Disulfiram** (CHEMBL964)	Drugs used in alcohol dependence	Glycolysis and fatty acid oxidation modulation	2	Q99714	HSD17B10	ACAA1, EHHADH
				P42858	HTT	GAPDH
**Haloperidol** (CHEMBL54)	Butyrophenone derivatives	Modulation of lipid metabolism	2	Q15125	EBP	TM7SF2
				Q99720	SIGMAR1	TM7SF2

PKM: Pyruvate kinase; EBP: 3-Beta-hydroxysteroid-Delta(8), Delta(7)-isomerase; SIGMAR1: Sigma non-opioid intracellular receptor 1; HSD17B10: 3-hydroxyacyl-CoA dehydrogenase type-2; HTT: Huntingtin; PGK1: phosphoglycerate kinase 1; GAPDH: glyceraldehyde 3-phosphate dehydrogenase; TM7SF2: Delta(14)-sterol reductase; ACAA1: 3-ketoacyl-CoA thiolase; EHHADH: peroxisomal bifunctional enzyme (enoyl-CoA hydratase and 3-hydroxyacyl CoA dehydrogenase).

**Table 2:** Ligand-binding and allosteric druggable cavities characterized in MSs associated with neurological drug targets.

Protein	Cavity Number	Pred. Max pKd	Drug Score	Druggability	Residues	Allosteric Cavities	Z-Score (>0.5)
P00558	1	11.01	1424	Strong	LEU:63, GLY:64, ARG:65, PRO:66, ASP:67, LYS:74, TYR:75, ARG:122, GLU:128, LY:166, THR:167, ALA:168, HIS:169, ARG:170, HIS:172, GLY:212, GLY:213, ALA:214, LYS:215, VAL:216, ALA:217, ASP:218, LYS:219, GLY:236, GLY:237, GLY:238, MET:239, PHE:241, SER:255, LEU:256, ASP:258, PHE:285, VAL:286, PHE:291, ASP:292, GLU:293, MET:311, GLY:312, LEU:313, ASP:314, CYS:315, ASN:336, GLY:337, PRO:338, VAL:339, GLY:340, VAL:341, PHE:342, GLU:343, TRP:344, PHE:347, THR:351, GLY:371, GLY:372, GLY:373, ASP:374, THR:375, ALA:376, THR:377, CYS:378, LYS:381, THR:393, GLY:394, GLY:395, GLY:396, ALA:397, SER:398, GLU:400	1	1.57
						2	1.26
						3	0.89
						4	0.79
						5	0.62
P04406	1	11.39	1477	Strong	GLY:82, PHE:83, GLY:84, ARG:85, ILE:86, GLY:87, ARG:88, ASP:121, THR:123, HIS:124, GLU:168, SER:169, THR:170, GLY:171, VAL:172, TYR:173, LEU:174, ILE:192, SER:193, ALA:194, PRO:195, SER:196, PRO:197, MET:201, ALA:222, SER:223, CYS:224, THR:225, THR:226, ASN:227, MET:247, THR:249, VAL:250, HIS:251, SER:252, TYR:253, THR:254, ALA:255, THR:256, GLN:257, LYS:258, PRO:263, SER:264, ARG:265, LYS:266, ALA:267, ASP:270, GLY:271, ILE:279, PRO:280, ALA:281, SER:282, THR:283, GLY:284, ALA:285, ALA:286, LYS:287, ALA:288, VAL:289, LYS:291, GLY:302, MET:303, ALA:304, PHE:305, ARG:306, THR:309, PRO:310, ASP:311, SER:313, TYR:386, ASN:388, GLU:389, TYR:392, SER:393, VAL:396	1	2.89
						2	0.94
Q08426	1	10.53	656	Strong	ASP:62, ILE:63, ARG:64, GLY:65, PHE:66, SER:67, ALA:68, LEU:129, LEU:259, LEU:260, GLN:261, SER:262, GLY:263, ALA:265, ARG:266, ALA:267, LEU:268, GLN:269, TYR:270, ALA:271, PHE:272, PHE:273, ALA:274, GLU:275, ARG:276, LYS:277, ALA:278, ASN:279, LYS:280, SER:642, GLU:644, ASP:647, PHE:665, LEU:709, LYS:710	1	1.84
P09110	1	9.48	3753	Strong	ARG:50, ALA:51, GLY:52, ASN:90, VAL:91, LEU:92, GLN:93, PRO:94, GLY:95, ASN:120, ARG:121, GLN:122, CYS:123, SER:124, SER:125, GLU:151, SER:152, MET:153, SER:154, LEU:155, ALA:156, ASP:157, ARG:158, GLY:159, ASN:163, ILE:164, THR:165, SER:166, LEU:168, ASP:176, CYS:177, LEU:178, ILE:179, PRO:180, MET:181, GLY:182, ILE:183, THR:184, ALA:263, PHE:264, THR:270, THR:271, ALA:272, GLY:273, SER:275, SER:276, GLN:277, VAL:278, SER:279, ASP:280, PRO:313, PRO:314, ASP:315, ILE:316, MET:317, ASN:345, GLU:346, ALA:347, PHE:348, VAL:373, HIS:377, PRO:378, LEU:379, CYS:408, ILE:409, GLY:410, THR:411, GLY:412, MET:413	-	-

## Data Availability

The original contributions presented in the study are included in the article/Supplementary Materials; further inquiries can be directed to the corresponding author/s.

## Supplementary Materials

The following supporting information can be downloaded at https://www.mdpi.com/article/10.3390/ph17050648/s1: Table S1: biological processes, Cellular components and Metabolic pathways terms enriched in proteins functionally related to Metabolic Switches; Table S2: OpenTargetDB association scores of neurodegenerative diseases to five MS indirectly regulated by ChEMBL nervous system drugs; Table S3: description of the 32 nervous system drugs associated with a single target in the PPI network; Table S4: metabolic Switches and natural ligands analyzed in the identification of druggable cavities; Table S5: enzymatic metabolic switches under PA (palmitic acid)-mediated cellular regulation.
