# Supplementary material for: Enzymatic Metabolic Switches of Astrocyte Response to Lipotoxicity as Potential Therapeutic Targets for Nervous System Diseases
Source: Pharmaceuticals (Basel). 2024 May 16;17(5):648. doi: 10.3390/ph17050648 (PMC11124372; doi:10.3390/ph17050648)
Supplement: Supplementary file 1 [file pharmaceuticals-17-00648-s001.zip › Supplementary Material.pdf]

Enzymatic metabolic switches of astrocyte response to lipotoxicity as potential therapeutic targets for neurodegenerative diseases

Pharmaceuticals - Supplementary Material

Supplementary tables

Table S1. Biological processes, Cellular components and Metabolic pathways terms enriched in proteins functionally related to Metabolic Switches.
Table S1 describes the enriched terms in the proteins functionally related to Metabolic Switches in Sec. 2.1.1.

The table S1 corresponds to the excel file "Table S1.xlsx"

Table S2. OpenTargetDB association scores of neurodegenerative diseases to five MS indirectly regulated by ChEMBL nervous system drugs.
Table S2 describes the neurodegenerative disease association scores for the five MSs mentioned in Sec. 2.1.2.

MSs UniProt ID	Related to neurodegenerative disease?	Open target database score*							
		OAS	GA	SM	D	PSB	TM	RE	AM
P00558	Yes	0.51328866	No data	No data	No data	0.843712939	0.012158616	No data	No data
P04406	Yes	0.552948973	No data	No data	No data	0.899017406	0.210833353	No data	No data
P09110	Yes°	0.074839921	No data	No data	No data	No data	0.615529933	No data	No data
O76062	Yes°	0.001478319	No data	No data	No data	No data	0.012158616	No data	No data
Q08426	-	-	No data	No data	No data	No data	No data	No data	No data

*According to the "neurodegenerative disease" score. If there was none (°), it corresponded to the nervous system disease with the highest score.
OAS: Overall Association Score, **GA**: Genetic Associations, **SM**: Somatic Mutations, **D**: drugs, **PSB**: Pathways Systems Biology, **TM**: Text Mining; **RE**: RNA Expression; **AM**: Animal Models.

Table S3. Description of the 32 nervous-system drugs associated with a single target in the PPI network.
Table S3 describes the 32 drugs with a single target in the DPI network in Sec. 2.1.2.

**Drug name** (CHEMBL ID)	**Drug related to neurodegenerative disease treatment**	**ID UniProt Target**	**Targets**	**Metabolic Switch Related**
CHEMBL95	Anticholinesterases	P05067	APP	GAPDH
CHEMBL1094	Other antiepileptics	P14618	PKM	PGK1, GAPDH
CHEMBL53	Dopamine agonist	Q99714	HSD17B10	EHHADH, ACAA1
CHEMBL1009	Dopa and dopa derivatives	Q99714	HSD17B10	EHHADH, ACAA1
CHEMBL1201236	Anti-Parkinson drugs	Q99714	HSD17B10	EHHADH, ACAA1
CHEMBL415	Non-selective monoamine reuptake inhibitors	Q99720	SIGMAR1	TM7FS2
CHEMBL41	Selective serotonin reuptake inhibitors	Q99720	SIGMAR1	TM7FS2
CHEMBL49	Azaspirodecanedione derivatives	Q99720	SIGMAR1	TM7FS2
CHEMBL479	Phenothiazines with piperidine structure	Q99720	SIGMAR1	TM7FS2
CHEMBL71	Phenothiazines with aliphatic side-chain	Q99720	SIGMAR1	TM7FS2
CHEMBL502	Anticholinesterases	Q99720	SIGMAR1	TM7FS2
CHEMBL14376	Other antipsychotics	Q99720	SIGMAR1	TM7FS2
CHEMBL549	Selective serotonin reuptake inhibitors	Q99720	SIGMAR1	TM7FS2
CHEMBL564	Phenothiazines with aliphatic side-chain	Q99720	SIGMAR1	TM7FS2
CHEMBL596	Opioid anesthetic/ Phenylpiperidine derivatives	Q99720	SIGMAR1	TM7FS2
CHEMBL621	Other antidepressants	Q99720	SIGMAR1	TM7FS2
CHEMBL629	Non-selective monoamine reuptake inhibitors	Q99720	SIGMAR1	TM7FS2
CHEMBL22242	Benzamides	Q99720	SIGMAR1	TM7FS2
CHEMBL660	Adamantane derivatives	Q99720	SIGMAR1	TM7FS2
CHEMBL698	Esters of aminobenzoic aci	Q99720	SIGMAR1	TM7FS2
CHEMBL716	Diazepines, oxazepines, thiazepines and oxepines	Q99720	SIGMAR1	TM7FS2
CHEMBL726	Phenothiazines with piperazine structure	Q99720	SIGMAR1	TM7FS2

CHEMBL728	Phenothiazines with piperazine structure	Q99720	SIGMAR1	TM7FS2
CHEMBL30008	Antivertigo preparations	Q99720	SIGMAR1	TM7FS2
CHEMBL809	Selective serotonin reuptake inhibitors	Q99720	SIGMAR1	TM7FS2
CHEMBL814	Selective serotonin reuptake inhibitors	Q99720	SIGMAR1	TM7FS2
CHEMBL43064	Antivertigo preparation	Q99720	SIGMAR1	TM7FS2
CHEMBL52440	Other nervous system drugs	Q99720	SIGMAR1	TM7FS2
CHEMBL1490	Tertiary amines	Q99720	SIGMAR1	TM7FS2
CHEMBL485253	Ergot alkaloids	Q99720	SIGMAR1	TM7FS2
CHEMBL1201203	Ethers of tropine or tropine derivatives	Q99720	SIGMAR1	TM7FS2
CHEMBL1628227	Non-selective monoamine reuptake inhibitor	Q99720	SIGMAR1	TM7FS2

APP: Amyloid-beta precursor protein; **PKM:** Pyruvate kinase; **HSD17B10:** 3-hydroxyacyl-CoA dehydrogenase type-2; **SIGMAR1:** Sigma non-opioid intracellular receptor 1; **GAPDH:** Glyceraldehyde 3-phosphate dehydrogenase; **PGK1**: Phosphoglycerate kinase 1; **EHHADH:** Peroxisomal bifunctional enzyme (Enoyl-CoA hydratase and 3-hydroxyacyl CoA dehydrogenase); **ACAA1:** 3-ketoacyl-CoA thiolase; **TM7FS2:** Delta(14)-sterol reductase.

Table S4. Metabolic Switches and natural ligands analyzed in the identification of druggable cavities.
Table S4 gives information about protein and ligand data employed in Sec. 2.1.3.

Protein							Natural Ligand			
**UniProt ID**	**Symbol**	**Name**	**Type**	**ID PDB**	**Coverage**	**Quality**	**Code**	**Name**	**Type**	**PDB ID ***
P00558	PGK1	*Phosphoglycerate kinase 1*	Exp	2WZB	100%	1.5	3PG	*3-phosphoglyceric acid*	CC	2WZB
P04406	GAPDH	*Glyceraldehyde-3-phosphate dehydrogenase*	Exp	3H9E	83%	1.7	G3P	*Glycealdehide-3-phosphate*	P	3LC2-P
Q08426	EHHADH	*Peroxisomal bifunctional enzyme (Enoyl-CoA hydratase and 3-hydroxyacyl CoA dehydrogenase)*	AF	-	-	-	HSC	*(s)-3-hydroxydecanoyl-coa*	P	5OMO
P09110	ACAA1	*3-ketoacyl-CoA thiolase, peroxisomal*	Exp	2IIK	93%	2.5	CoA	*Coenzyme A*	P	2WKV-B
O76062	TM7SF2	*Delta(14)-sterol reductase*	AF	-	-	-	NDP	*(NADPH) Dihydro-nicotinamide-adenine-dinucleotide phosphate*	P	4QUV

CC: Natural ligand co-crystallized in PDB; **P:** Natural ligand predicted by COACH-D.
* PDB ID correspond to complex of the MSs with their natural ligand in PDB for CC and template complex for ligand-binding site prediction in COACH-D.

Table S5. Enzymatic metabolic switches under PA (palmitic acid)-mediated cellular regulation.

Reaction ID	Biological subsystem	ID UniProt	Name	*Biological importance
GHMT2r	*Glycine, serine, alanine, and threonine metabolism*	P34896	*Serine hydroxymethyltransferase*	Reaction associated with the polyglutamylation of folate. It is also fully and directionally coupled to purine, serine, alanine, and threonine metabolism.
r0735	*Fatty acid oxidation*	Q9BY49	*Peroxisomal trans-2-enoyl-CoA reductase*	Fatty acid oxidation
r0276	*Purine catabolism*	P36959	*Guanosine monophosphate reductase*	catalyzes the NADPH-dependent deamination of guanosine monophosphate to inosinic acid
r0276	*Purine catabolism*	Q9P2T1	*Guanosine monophosphate reductase 2*	catalyzes the NADPH-dependent deamination of guanosine monophosphate to inosinic acid
HMGCOASim	*Cholesterol metabolism*	P54868	*Hydroxymethylglutaryl-CoA synthase*	It catalyzes the reaction associated with the condensation of acetyl-CoA with acetoacetyl-CoA to form 3-hydroxy-3-methylglutaryl-CoA (HMG-CoA).
PGK	*Glycolysis/gluconeogenesis*	P00558	*Phosphoglycerate kinase 1*	Catalysis of the reversible transfer of a phosphate group from 1,3-bisphosphoglycerate to ADP producing 3-phosphoglycerate and ATP
GAPD	*Glycolysis/gluconeogenesis*	O14556	*Glyceraldehyde-3-phosphate dehydrogenase*	Catalysis of glyceraldehyde 3-phosphate to d-glycerate 1,3-bisphosphate
FTHFLm	*Folate metabolism*	P11586	*C-1-tetrahydrofolate synthase, cytoplasmic*	Formate-tetrahydrofolate ligase reaction, which participates in the transfer of carbon units, essential for several biosynthetic pathways.
GAPD	*Glycolysis/gluconeogenesis*	P04406	*Glyceraldehyde-3-phosphate dehydrogenase*	catalysis of glyceraldehyde 3-phosphate to d-glycerate 1,3-bisphosphate
FAOXC16080x	*Fatty acid oxidation*	P09110	*3-ketoacyl-CoA thiolase, peroxisomal*	β-oxidation of long-chain acids
r0735	*Fatty acid oxidation*	Q9BV79	*Enoyl-[acyl-carrier-protein] reductase, mitochondria*	Fatty acid oxidation
r0839	*Transport, extracellular*	O76062	*Delta(14)-sterol reductase TM7SF2*	Orotate transport (r0839) for the biosynthesis of RNA pyrimidines
PGK	*Glycolysis/gluconeogenesis*	P07205	*Phosphoglycerate kinase 2*	catalysis of the reversible transfer of a phosphate group from 1,3-bisphosphoglycerate to ADP producing 3-phosphoglycerate and ATP

Supplementary Material

FAOXC16080x	*Fatty acid oxidation*	Q08426	*Peroxisomal bifunctional enzyme (Enoyl-CoA hydratase and 3-hydroxyacyl CoA dehydrogenase)*	β-oxidation of long-chain acids
FAOXC8C6x	*Fatty acid oxidation*	P51659	*Peroxisomal multifunctional enzyme type 2*	β-oxidation of fatty acids C8- > C6
FAOXC6C4x	*Fatty acid oxidation*	Q15067	*Peroxisomal acyl-coenzyme A oxidase 1*	β-oxidation of fatty acids C6- > C4
